# Neutrophil-to-Lymphocyte Ratio (NLR) Useful as a Cost-Effective Preliminary Prognostic Marker in ST-Elevation Myocardial Infarction (STEMI): An Observational Study From a Tertiary Care Hospital in Northeast India

**DOI:** 10.7759/cureus.36885

**Published:** 2023-03-29

**Authors:** Dibya J Sharma, Hirak J Nath, Akash Batta, Ashok K Goala

**Affiliations:** 1 Medicine and Gastroenterology, Silchar Medical College and Hospital, Silchar, IND; 2 Medicine, Silchar Medical College and Hospital, Silchar, IND; 3 Cardiology, Dayanand Medical College and Hospital, Ludhiana, IND

**Keywords:** mortality, diabetes, preliminary, neutrophil, observational, biomarker, inflammation, nlr, stemi, myocardial infarction

## Abstract

Introduction

Myocardial infarction, a major consequence of coronary artery disease, is an important cause of in-hospital mortality and morbidity worldwide. Blood neutrophil-to-lymphocyte ratio (NLR) is a novel laboratory marker of systemic inflammation that can predict the severity and mortality in various non-cardiovascular illnesses, including malignancy and infective pathology. We sought to evaluate its potential in predicting the outcome in hospitalized patients with myocardial infarction.

Material and methods

The index study was conducted at Silchar Medical College and Hospital from June 1, 2021 to May 31, 2022, with the aim of evaluating the role of NLR in determining the outcomes of ST-elevation myocardial infarction (STEMI). A total of 110 patients fulfilling the requisite criteria and admitted to the cardiology and medicine departments of the hospital with evidence of STEMI were included in the study and evaluated for the relationship of NLR with various outcome variables in STEMI.

Results

Out of 110 patients, 69.1% were males. The mean age of the study population was 58.2±15.3 years. The baseline characteristics and risk factors of patients who survived the acute attack of STEMI and those who died from complications of STEMI were similar. Laboratory parameters which correlated with worse outcomes included a higher fasting triglyceride level (173.4 mg vs. 215.6 mg, p < 0.001), a higher blood neutrophil count at baseline, 24 hours and 72 hours (70.1% vs. 69.04%, 66.3% vs. 75.2%, 81.6% vs. 73.8%, p<0.05), a higher NLR value at baseline, 24 hours and 72 hours (2.91 ± 1.13 vs. 3.19 ± 2.32, 2.39 ± 0.74 vs. 5.56 ± 4.11, 5.1 ± 4.38 vs. 3.01 ± 1.02, p < 0.05). Among patients hospitalized with STEMI who had high NLR, had significantly elevated incidence of complications, including a higher acute, left ventricular failure (42.8% vs. 35.9%; p < 0.05) as well as increased risk of mortality (66.7% vs. 33.3%; p < 0.05) compared to low NLR group.

Conclusion

NLR can predict the outcome among STEMI patients in terms of morbidity and mortality and correlates with poor left ventricular function. NLR can serve as a potential tool for early identification and efficient triage of STEMI patients during initial presentation to the ED. Its utility is more so in resource-constrained developing countries with limited access to health care. The significant advantage of NLR is its easy accessibility, rapid turnaround time, and inexpensiveness.

## Introduction

Coronary artery disease is a spectrum of disorders contributing to most of the myocardial infarction and heart failure incidences and is considered the leading cause of mortality worldwide [1]. Atherosclerosis of coronary arteries which takes decades to manifest clinically, is the primary predisposing pathologic factor responsible for the development of coronary heart disease. A complex immune and inflammatory pathophysiological process is thought to be crucial for the beginning and development of atherosclerotic plaques that ultimately leads to the clinical manifestation of the disorder [2]. Although numerous inflammatory markers, including troponin T/I, lactate dehydrogenase (LDH), and creatine kinase (CK-MB), are linked to worsened clinical outcomes in both ST elevation and non-ST elevation myocardial infarction (NSTEMI), there is an unmet need for a cost-effective biomarker for impoverished countries of the world. The neutrophil-to-lymphocyte ratio (NLR), which is a relatively cheap inflammatory marker, can act as a bridge to mitigate the gap in assessing the cardiovascular risk and outcomes in patients with acute myocardial infarction [3]. Recent data indicates a strong independent relationship between increased complications after acute myocardial infarction in patients with a high NLR at admission and the initial post-hospitalization period. However, limited data are available on the Indian population, which is quite different from the western population as they have an early age of onset and a rapid progression. Accordingly, we sought to assess the predictive value of NLR in ST-elevation myocardial infarction (STEMI) among patients from northeast India with distinct ethnicity and unique genetic compositions [4-7]. The index study was undertaken with the aim of assessing the level of NLR in patients with STEMI and correlating its association with myocardial dysfunction manifested by ejection fraction (EF) on 2D-echocardiography or cardiac damage as measured by CK-MB level in blood.

## Materials and methods

The index study is a prospective observational study where 110 consecutive patients admitted to the ICU and general ward of the departments of cardiology and internal medicine at Silchar Medical College and Hospital with STEMI were selected after fulfilling the inclusion and exclusion criteria. The study was conducted over one year period from June 2021 to May 2022. STEMI was diagnosed in patients with typical chest pain history and ECG showing typical STEMI. The primary outcome of the study was the in-hospital mortality of patients with STEMI. In all the patients, total WBC count, neutrophil, and lymphocyte monocyte counts were calculated from venous blood collected during hospitalization. NLR was calculated as total neutrophil counts divided by total lymphocyte count and was calculated from the absolute values of neutrophil and lymphocyte from the same venous blood collected at admission. The NLR was calculated for every patient and was correlated with in-hospital mortality. Statistical analysis was prepared using Microsoft Excel and SPSS for Windows, version 20.0 (SPSS Inc., Chicago). The Institutional Ethics Committee approved the study after a thorough review (SMC/12/ 2022), and written informed consent was taken from all the patients.

Inclusion and exclusion criteria

Patients with age ≥13 years of either sex, with either increase in serum cardiac biomarkers or ECG showing STEMI, were included in the study. Patients presenting with NSTEMI and unstable angina were excluded. Patients with any of these associated conditions that can affect NLR, including inflammatory conditions such as collagen-vascular disorders, acute or chronic infectious diseases, auto-immune and neoplastic diseases, chronic hepatic diseases, renal failure, thyroid disorders, previous valvular heart disease and patients who did not give consent for the study were excluded. On initial admission, the diagnosis was confirmed using standard methods, and patients were treated as per the American College of Cardiology/American Heart Association (ACC/AHA) protocols followed in our institution.

## Results

Demographic profile

The total study population included in our study was 110, out of which 69.1% (n=76) were males and 30.1% patients were females (n=34). Maximum number of patients belonged to the age group of 50-70 years. Baseline characteristics of the patients who were hospitalized with STEMI were similar among the survival and non-survival group (Table 1). The mean age of the study population among survivors was 58.78 years, whereas among the non-survivor group, it was 54.89 years (p >0.05). The majority of the patients were male, with 67.4% in the survival group and 66.7% in the non-survival group, which could be attributed to the higher representation of risk factors of STEMI among the male population. Similarly, the impact of gender, smoking, alcohol, diabetes mellitus, and hypertension on the incidence of mortality in the study population was insignificant (p>0.05). Baseline characteristics of patients who were discharged alive (survivor) or deceased (non-survivor) are represented in Table 1.

**Table 1:** Baseline characteristics of survivor and non-survivor patients.

Characteristics	Survivor	Non-survivor	P-value
Mean age	58.78 ± 15.3	54.89 ± 18.33	0.342
Gender (Males, %)	67.4	66.7	0.952
Smoking	51.1	61.1	0.436
Alcohol	40.2	27.8	0.32
Diabetes mellitus	56.5	72.2	0.215
Hypertension	70.7	72.2	0.893

Laboratory parameters

When the laboratory parameters of patients with STEMI were analyzed, it was detected that the mean fasting triglyceride level (215.61mg/dL) among deceased subjects was significantly higher as compared to the survivors (173.46 mg/dL), implicating the pivotal role of dyslipidemia in the pathogenesis of acute STEMI. Patients who had high blood sugar levels (random blood sugar [RBS] >150 mg/dL) at the time of admission had poorer outcomes as compared to the patients who had lower blood sugar levels (RBS < 150 mg/dL) during hospitalization. This finding was statistically significant, suggesting the role of stress hyperglycemia as a useful predictor of the major adverse cardiac events in STEMI patients with elevated NLR. Blood neutrophil counts at admission, at 24 hours, and 72 hours after admission was assessed and were found to be higher (significant) in the deceased population (75.28 ± 7.19, 81.61 ± 8.96, 73.89 ± 7.58) as compared to the patients who had survived (70.18 ± 74.21, 69.04 ± 11.63, 66.34 ± 6.670) the acute attack (p < 0.05). On the contrary, lymphocyte counts done at admission, at 24 hours, and 72 hours after admission were significantly higher in the patients who had survived (27.84 ± 10.52, 27.84 ± 10.52, 29.36 ± 5.82) the acute attack as compared to the patients who succumbed (21.2 ± 7.19, 15.39 ± 8.96, 23.11 ± 7.58) to the illness (p < 0.05). Three consecutive values of NLR were calculated, and the value among the deceased patients (5.56 ± 4.11, 5.1 ± 4.38, 3.01 ± 1.02) was found to be higher significantly as compared to the patients who survived the ischemic event (2.91 ± 1.13, 3.19 ± 2.32, 2.39 ± 0.74, p < 0.05). Comparative laboratory parameters of survivors and non-survivor patients are shown in Table 2.

**Table 2:** Laboratory parameters of survivor and non-survivor patients. NLR: Neutrophil-to-lymphocyte ratio; RBS: Random blood sugar.

Characteristics	Survivor (Approx.)	Non-survivor (Approx.)	P-value
Fasting triglyceride (mg/dL)	173.46	215.61	0.025
RBS at admission (mg/dL)	143.83	237.94	0.000
Baseline neutrophil count	70.18	75.28	0.015
Neutrophil count at 24 hours	69.04	81.61	0.000
Neutrophil count at 72 hours	66.34	73.89	0.000
Baseline lymphocyte count	26.61	21.2	0.011
Lymphocyte count at 24 hours	27.84	15.39	0.000
Lymphocyte count at 72 hours	29.36	23.11	0.000
Baseline total count	8675.11	11172.22	0.001
Total count at 24 hours	8359.46	9822.78	0.016
Total count at 72 hours	7202.28	8961.1	0.001
Baseline NLR	2.91 ± 1.13	5.56 ± 4.11	0.000
NLR at 24 hours	3.19 ± 2.32	5.1 ± 4.38	0.008
NLR at 72 hours	2.39 ± 0.74	3.01 ± 1.02	0.003

Complications

On analyzing the complications of STEMI associated with high NLR (NLR >3), it was noted that 80% (n=88) of the patients had developed major acute adverse cardiac events after an episode of STEMI (Table 3). The incidence of overall complication was higher among patients with high NLR (82.6%) as compared to those who died from the disease (66.7%) with low NLR (NLR<3), indicating that complications are more common with increased severity of inflammation of STEMI (p <0.05). A total of 9.1% of patients had episodes of arrhythmias after the acute myocardial infarction. However, the incidence of arrhythmia was higher in the elevated NLR group than in the low NLR group (7.6%), indicating a positive association between high NLR value and arrhythmias, although the relationship was statistically insignificant. On the other hand, LVEF <50%, which resonates with the development of low EF heart failure, was found to be significantly higher (p < 0.05) among the elevated NLR (42.77 ± 10.6) group as compared to the truncated NLR group (35.94 ± 7.03). The incidence of hypotension with systolic blood pressure (SBP) less than 90 mm Hg was more among patients with elevated NLR (17.4%) as compared to the patients who had low NLR (0%). However, it was not statistically significant (p> 0.05). The complications associated with STEMI in the high and low NLR groups have been compared in 

**Table 3:** Complications of STEMI with high and low NLR. STEMI: ST-elevation myocardial infarction (STEMI); NLR: Neutrophil-to-lymphocyte ratio; LVEF: Left ventricular ejection fraction.

Characteristics	High NLR	Low NLR	P-value
Overall complications	82.6	66.7	0.122
Arrhythmias	7.6	16.7	0.222
LVEF < 50%	42.77 ± 10.6	35.94 ± 7.03	0.01
Hypotension	17.4	0	0.056

Course in the hospital

Fifty percent (50%) of the patients with high NLR had more than three days of hospital stay, whereas 71.7% of the patients with low NLR had more than three days of hospitalization. However, this difference was not statistically significant (p < 0.05). This suggests that patients with higher NLR were associated with increased short-term fatality rates from STEMI. Similarly need for ICU admission was also noted in 83.3% of the patients who had elevated NLR, which was significantly higher as compared to the patients with low NLR (25%), indicating a severe disease course (p<0.05). Twelve patients (66.7%) had died from the complications of STEMI with higher NLR value, whereas only six patients (33.3%) had died in the low NLR group (p < 0.05). Correspondingly, most patients (60.9%) in the low NLR group could be discharged from the hospital. Only 39.1% (n=1) of patients with high NLR had survived the acute attack of STEMI and could be released from the hospital, which is statistically significant. Therefore STEMI patients with high NLR need to be triaged early for round-the-clock monitoring, and proactive therapy needs to be initiated early to decrease the mortality rate. The hospital course in STEMI with high and low NLR is compared in Table 4.

**Table 4:** Hospital course in STEMI with high and low NLR. STEMI: ST-elevation myocardial infarction; NLR: Neutrophil-to-lymphocyte ratio.

Characteristics	Low NLR	High NLR	P-value
Hospital stay <3 days	28.3	50.0	0.07
Hospital stay >3 days	71.7	50.0	
ICU admissions	25.0	83.3	0.000
Mortality	6 (33.3%)	12 (66.7%)	0.031
Discharged	56 (60.9%)	36 (39.1%)	0.031

## Discussion

The relationship between leukocytosis and acute STEMI is well known. A simple and widely accessible blood test like the NLR can provide information about the complexity and severity of the plaque burden at the time of admission. NLR can also be beneficial in assessing the severity of acute coronary syndrome in terms of clinical outcomes over and above leukocytosis alone, which is largely non-specific. NLR is an easily measurable biomarker with a positive correlation with systemic inflammation. A high NLR in blood is associated with increased hospital mortality and complications after STEMI [8]. This portrays the role of neutrophils as the first line of defense of the innate immune system, which is recruited to the ischemic region, where they initiate the inflammatory response. In a study on the importance of the prognostic value of NLR in patients with STEMK by Park JS et al. in 2018, which included 326 patients, the mean NLR was 4.7 ±5.2. Similarly, the index study, which included 110 patients from the northeastern part of India, had a mean NLR value of 3.5, possibly due to contrasting genetic makeup and distinct response to inflammation. The all-cause mortality occurred in 16% (n=18) patients in our study, which is comparable to 14% (n=46) patients in the study done by Park JS et al. Initial NLR at admission in the study done by Park JS et al. (6.39±89 vs. 4.2±3.1, p< 0.05) was similarly echoed in our study (5.56 ±4.11 vs. 2.91±1.13, p<0.05), emphasizing the role of inflammation in the initiation of ischemia. In the multivariate regression model, the higher NLR was independently associated with an increased rate of mortality (p < 0.05), as shown in the study by Park JS et al. [9]. Similarly, Yoon GS et al. in 2021 showed that patients in the high NLR group (NLR>4.90) had a higher risk of mechanical complications of STEMI (p < 0.05) as compared with low and intermediate NLR groups (13% vs. 13% vs. 23%). On the other hand, our study, which divided the study population based on a cut-off NLR value of 3 (NLR <3 and NLR >3), found that the overall complications were higher in the group having NLR>3 than in the low NLR group (82.6% vs. 66.7%, p< 0.05). On multivariable analysis, NLR remained an independent risk factor for left ventricular dysfunction (p < 0.05) [10]. In our study, similar results were detected with NLR as a predictor of decreased LVEF (EF<50%) among STEMI patients (p < 0.05). All the patients recruited and analyzed were STEMI patients. The high NLR group had a significantly increased incidence of low LVEF (<50 %) compared to the low NLR group among all the STEMI patients included in the trial. However, elevated total leukocyte count, total neutrophil count, NLR, and plasma glucose (stress hyperglycemia) on admission have been found to be associated with increased incidence of major cardiovascular events and death in hospitals in patients with STEMI. Stress hyperglycemia at admission is associated with elevated plasma IL 18, CRP level, cytotoxic T cells, CD16+/ CD 56+ percentage, and CD4/CD8 ratio while inhibitors of T-cell activation like CD152 level is reduced implicating the role of inflammation in limiting the immune response of ischemia in STEMI. Blood NLR is a simple, cheap, and easily available alternative marker of inflammation that can be used in the prognostic stratification of STEMI patients at hospitalization [10-12]. Evidence from our study and other similar studies points to its practical relevance as an affordable, easily accessible, and reliable marker with rapid turnaround time which is the need of the hour while managing STEMI patients. In resource-constrained countries like ours this is more likely to find its place where access to other biomarkers which are expensive and take longer turnaround times may not be possible. Hence, in the future, it may find a place along with other markers in the early and efficient triage of patients, especially in resource-constrained settings.

A higher NLR is related to lower EF and higher long-term mortality in STEMI patients but fewer rates of hospital complications. However, no significant differences in NLR were detected among patients with or without atrial fibrillation (AF) and those with ventricular tachycardia (VT)/ventricular fibrillation (VF) beyond the first day. However, NLR was significantly higher in patients with VT/VF within the first day [13-14]. On the contrary, studies have demonstrated a correlation between NLR (NLR ≥ 3.29) and mean platelet volume to lymphocyte ratio (MPVLR ≥ 8.57) with the development of AF in patients presenting with STEMI (p<0.05). Elevated NLR and MPVLR values in patients with BMI more than 24.1± 5.6 kg/m2 and NYHA grade IV heart failure were found to be risk factors for poor outcomes in STEMI [15]. This dissimilarity in outcome could be due to different ethnic backgrounds and underlying risk factors of the study population.

A higher NLR is related to lower EF and higher all-cause mortality in STEMI patients undergoing primary percutaneous coronary intervention PCI, which was demonstrated in patients presenting with STEMI in previous studies. NLR is independently associated with lower EF and higher mortality rates up to five years of initial presentation. NLR value appears additive to conventional risk factors and commonly used biomarkers [13]. An increased red cell distribution width (RDW) and elevated NLR on admission in patients of anterior STEMI treated with primary PCI are associated with LV systolic dysfunction [16].

NLR can predict mortality and major adverse cardiac events in acute coronary syndrome. A recent systematic review and meta-analysis showed that in patients with a recent ACS, an elevated pre-treatment NLR value was effective in predicting the risk of mortality/major adverse cardiovascular events (MACEs). NLR can serve as an inexpensive and useful marker with strong prognostic significance in ACS patients, especially when patients have a high NLR value [17].

According to Ayca B et al., a high pre-procedural NLR was associated with an increased incidence of stent thrombosis and overall mortality in STEMI patients [18]. NLR based on an optimal cut-off value of 7.4 can be used as an excellent predictor of short and long-term survival in patients with revascularized STEMI, which warrant confirmation as a prognostic indicator with larger scale multi-center prospective studies in the future [19].

Summary

In this single-center observational study, NLR was used to prognosticate the patients with STEMI. NLR was compared between survivors and non-survivors, and it was found that NLR was a practical and cost-effective parameter that can predict the development of arrhythmia, hypotension, and in-hospital mortality. This study substantiates the previous conclusions of existing literature. 

Drawback of the study

The study population was relatively small and non-randomized. A randomized controlled trial (RCT) involving a larger population and multiple centers can be helpful in better delineating the study objective. Moreover, a correlation between NLR and existing prognostic markers of STEMI, such as CK-MB, CRP, and troponin-I, was not done, which could have further illuminated the utility of NLR in clinical practice. None of our patients has undergone PCI, as it is not available in our setup. 

## Conclusions

The unmet need for an early biomarker that can be useful in the triage of patients with myocardial infarction in underdeveloped countries can be accomplished by inflammatory markers like NLR. NLR can prognosticate patients with STEMI in terms of the development of complications like arrhythmia, hypotension, need for ICU admission, decreased cardiac ejection fraction, and mortality. Blood NLR value can be used as a cost-effective and readily available marker in the prognostication of STEMI patients during hospitalization. However, a larger multicentre RCT is required before NLR's role in STEMI is comprehensibly established. 
